# Southampton PRegnancy Intervention for the Next Generation (SPRING): protocol for a randomised controlled trial

**DOI:** 10.1186/s13063-016-1603-y

**Published:** 2016-10-12

**Authors:** Janis Baird, Mary Barker, Nicholas C. Harvey, Wendy Lawrence, Christina Vogel, Megan Jarman, Rufia Begum, Tannaze Tinati, Pamela Mahon, Sofia Strommer, Taylor Rose, Hazel Inskip, Cyrus Cooper

**Affiliations:** 1 MRC Lifecourse Epidemiology Unit, University of Southampton, Southampton, SO16 6YD UK; 2 NIHR Nutrition Biomedical Research Centre, University of Southampton, Southampton, SO16 6YD UK; 3 Li Ka Shing Centre for Health Research Innovation, Department of Agriculture, Food and Nutritional Science, University of Alberta, Edmonton, T6G 2E1 Alberta Canada

## Abstract

**Background:**

The nutritional status and health of mothers influence the growth and development of infants during pregnancy and postnatal life. Interventions that focus on improving the nutritional status and lifestyle of mothers have the potential to optimise the development of the fetus as well as improve the health of mothers themselves. Improving the diets of women of childbearing age is likely to require complex interventions that are delivered in a socially and culturally appropriate context. In this study we aim to test the efficacy of two interventions: behaviour change (Healthy Conversation Skills) and vitamin D supplementation, and to explore the efficacy of an intervention that combines both, in improving the diet quality and nutritional status of pregnant women.

**Methods/design:**

Women attending the maternity hospital in Southampton are recruited at between 8 and 12 weeks gestation. They are randomised to one of four groups following a factorial design: Healthy Conversation Skills support plus vitamin D supplementation (1000 IU cholecalciferol) (*n* = 150); Healthy Conversation Skills support plus placebo (*n* = 150); usual care plus vitamin D supplementation (*n* = 150); usual care plus placebo (*n* = 150). Questionnaire data include parity, sunlight exposure, diet assessment allowing assessment of diet quality, cigarette and alcohol consumption, well-being, self-efficacy and food involvement. At 19 and 34 weeks maternal anthropometry is assessed and blood samples taken to measure 25(OH) vitamin D. Maternal diet quality and 25(OH) vitamin D are the primary outcomes. Secondary outcomes are women’s level of self-efficacy at 34 weeks, pregnancy weight gain, women’s self-efficacy and breastfeeding status at one month after birth and neonatal bone mineral content, assessed by DXA within the first 14 days after birth.

**Discussion:**

This trial is evaluating two approaches to improving maternal diet: a behaviour change intervention and vitamin D supplementation. The factorial design of this trial has the advantage of enabling each intervention to be tested separately as well as allowing exploration of the synergistic effect of both interventions on women’s diets and vitamin D levels.

**Trial registration:**

ISRCTN07227232. Registered on 13 September 2013.

## Background

The nutritional status and health of mothers influence the growth and development of infants during pregnancy and postnatal life [1]. Growth and development at these stages of the lifecourse will influence the risk of non-communicable diseases such as cardiovascular disease and obesity in adulthood. Interventions that focus on improving the nutritional status and lifestyle of mothers have the potential to optimise the growth and development of the fetus as well as improve the health of mothers themselves. Studies have shown that maternal nutrition influences child body composition [2, 3]. There are two principal approaches to improving the nutritional status of pregnant women: nutritional supplementation (multiple-micronutrient supplementation as well as single vitamin supplements to correct deficiencies) or behaviour change interventions that aim to improve the diet quality of pregnant women [4]. Improving the diets of women of childbearing age is likely to require complex interventions that are delivered in a socially and culturally appropriate context [5].

Multiple-micronutrient approaches have improved maternal and birth outcomes in studies of pregnant women in developing countries [6]. In Mumbai, India, for example, a food-based multiple-micronutrient supplement started preconceptionally and given throughout pregnancy halved the prevalence of maternal gestational diabetes [7]. Correction of specific vitamin deficiencies during pregnancy has improved outcomes for pregnant women and their babies in developed and developing country settings [8]. In a recent UK multi-centre trial of vitamin D supplementation during pregnancy, the Maternal Vitamin D Osteoporosis Study (MAVIDOS), supplementation corrected maternal vitamin D deficiency and optimised infant levels of vitamin D [9, 10].

Behaviour change approaches during pregnancy have the potential to improve the health behaviours of pregnant women and, whereas nutrient supplementation addresses specific nutrient deficiencies, behaviour change approaches can improve overall diet quality. Most pregnant women want to do their best for their baby, and so pregnancy presents an opportunity to tackle unhealthy lifestyle choices, such as smoking, and to promote healthy ones, such as intention to breastfeed [11]. Women’s confidence or self-efficacy that they can make such changes is an important determinant of whether their health behaviours will improve [12]. Low levels of self-efficacy are a barrier to healthful eating among women from disadvantaged backgrounds [13], and many studies have demonstrated a relationship between higher levels of self-efficacy and better dietary behaviours [14]. Reviews of evidence have provided useful insights into the features of behaviour change interventions associated with effectiveness in women of childbearing age and disadvantaged groups: providing information on risks and benefits of health behaviours combined with goal setting and continued support after the initial intervention were more likely to lead to behaviour change [15, 16].

The evidence points to the need for empowerment approaches that work by improving the self-efficacy of participants. We applied such an approach to an intervention which aimed to improve the health behaviour of women from disadvantaged backgrounds, a group in which there is an established link between low self-efficacy and poor quality diet [13, 17]. The intervention, the Southampton Initiative for Health (SIH), aimed to improve the diets of women from disadvantaged backgrounds. The intervention involved training Sure Start Children’s Centre staff, who work with women and children from disadvantaged families, in skills to support behaviour change [18]. The training led to changes in the way the staff interacted with women and, one year after training, staff in the intervention area were still using these skills and were using them significantly more than staff in the control area [19]. Evaluation showed that the intervention had a protective effect on the sense of control and self-efficacy of women who came into contact with trained staff, intermediate factors on the causal pathway between exposure to the intervention and change in diet and physical activity, though an influence on diet was not observed [20]. These findings suggested that the intervention has the potential to improve health behaviours in settings that offer women greater and more frequent exposure to trained staff. Pregnancy and childbirth offer such a setting.

Evidence from two recent UK trials supports the potential of behaviour change interventions during pregnancy to improve the healthfulness of women’s diets. In the UPBEAT and LIMIT trials, women received regular support from health care workers throughout their pregnancies. In LIMIT, the intervention was informed by stage theories of health decision making and comprised advice on behavioural strategies for healthful eating and increased physical activity that was delivered by a research dietician and trained research assistants. Women were encouraged to set diet and exercise goals and to self-monitor their progress towards these goals [21]. In UPBEAT, the intervention was informed by social cognitive theory. Health trainers delivered eight weekly sessions in which women received advice on improving diet and physical activity through a range of strategies, including identification of barriers to change, setting of SMART goals and self-monitoring [22]. These behaviour change interventions led to improvements in diet, although they did not improve the primary outcomes of gestational diabetes and babies born large for gestational age [21, 22]. Importantly, both interventions adopted empowerment approaches, suggesting that such an approach is likely to be successful in bringing about behaviour change.

Given that both supplementation and behaviour change intervention approaches have led to improvements in the diets of pregnant women, it is possible that interventions that combine both approaches might have a greater impact on diet than when each is delivered on its own. Evidence of the importance of self-efficacy as a predictor of women’s diets indicates that interventions that are effective in improving maternal diet will need to take account of women’s social circumstances and the barriers to healthy eating that they experience in their daily lives. This suggests that complex multi-component interventions will be required to bring about the sort of changes in women’s diets that are important for the health of their babies and has led us to assess such a multi-component intervention using a factorial design.

In the Southampton PRegnancy Intervention for the Next Generation (SPRING) trial, we have combined a behaviour change approach with vitamin D supplementation. We believe that this approach to improving health behaviour through enhancement of women’s self-efficacy has the potential to address barriers to taking supplements during pregnancy as well as to improve overall dietary quality.

### Aims

There are three aims of this trial. The first is to assess the efficacy of a behaviour change intervention (Healthy Conversation Skills) in improving the diet quality of pregnant women. The second is to assess the efficacy of oral daily vitamin D supplementation in improving the vitamin D status of pregnant women. The third is to explore the efficacy of an intervention combining vitamin D supplementation and behaviour change support in improving the diet quality and nutritional status of pregnant women.

### Study design

The study is a randomised controlled trial that uses a two-by-two factorial design. The two interventions being tested are exposure to nurses trained in skills to support behaviour change (Healthy Conversation Skills) and daily oral vitamin D supplementation (1000 IU cholecalciferol daily). The women taking part in the study and the research nurses delivering the intervention are blind to the vitamin D intervention, so this part of the trial is double blind. However, the same is not possible for the behaviour change intervention. There are four groups of study participants:

- Healthy Conversation Skills support plus vitamin D supplementation
- Healthy Conversation Skills support plus placebo
- Usual care plus vitamin D supplementation
- Usual care plus placebo.

### Primary outcomes

Given the factorial design of the trial, there are two primary outcomes:

- Maternal circulating plasma 25(OH) vitamin D concentration at 34 weeks
- Maternal diet quality at 34 weeks gestation assessed with a 20-item food frequency questionnaire [23]

### Secondary outcomes

The secondary outcomes are:

- Women’s level of self-efficacy at 34 weeks, assessed with the General Self-Efficacy scale [24]
- Pregnancy weight gain assessed by gain in weight between recruitment and 34 weeks gestation
- Maternal diet quality one month after birth
- Breastfeeding status one month after birth (yes/no)
- Neonatal whole body bone mineral content (BMC), lean and fat mass within 2 weeks after birth assessed by dual-energy X-ray absorptiometry (DXA).

## Methods/design

**Fig. 1:**
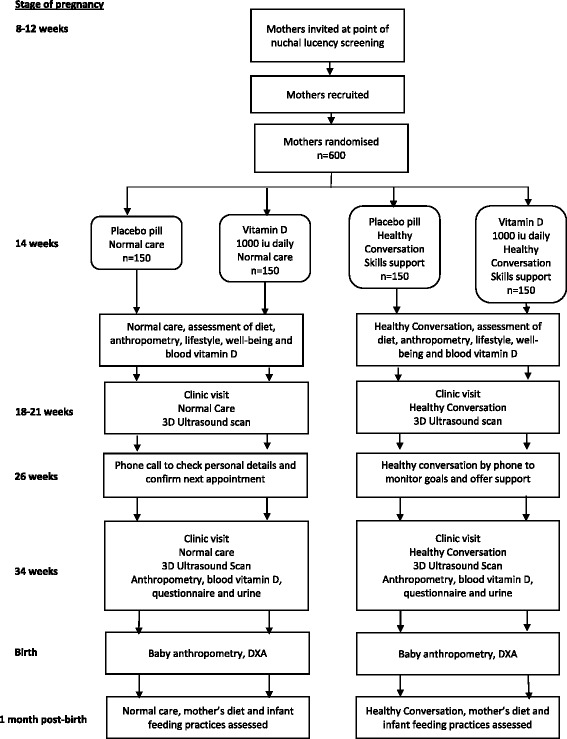
Trial summary: Southampton PRegnancy Intervention for the Next Generation (SPRING)

The trial procedures are summarised in Fig. 1.

### Study setting

The setting for the trial is the Princess Anne Maternity Hospital, Southampton, UK. Southampton is a relatively deprived city on the south coast of England and is among the 25 % most deprived local authorities within the UK based on the Index of Multiple Deprivation. Princess Anne Hospital is the setting for the majority of births within the city and provides maternity care for 5000 women each year.

### Recruitment at 8–12 weeks

Women are recruited to the study in early pregnancy, and by around 12 weeks if possible, using an approach that has been successful in the MAVIDOS trial of vitamin D supplementation during pregnancy [9]. Posters in general practitioner (GP) surgeries and in the maternity hospital waiting area publicise the study. Each woman booked for her antenatal care at Princess Anne Hospital is sent an information leaflet outlining the study along with the routine letter she receives inviting her to her nuchal translucency or early dating scan. Research nurses approach women who are attending the Princess Anne Hospital for their nuchal translucency or dating scan between 8 and 12 weeks gestation and invite them to discuss the study, having first checked whether they have read the SPRING information leaflet. If women agree to take part, then the nurses check their eligibility for the study and take them through the consent process. An interpreter can be involved if required.

Women who decline to take part in the study are given an information sheet explaining that we are running a sub-study about why women do not take part in research. They are asked whether they would be willing to complete a one-page questionnaire giving details about their age, educational attainment and number of children, and to indicate the reasons why they declined to take part in the study. It is made clear to women that they are under no obligation to complete the questionnaire. They are also asked whether they would be willing to take part in a 30-minute interview with a researcher to further explore their reasons for declining.

### Healthy Conversation Skills support

Healthy Conversation Skills training is underpinned by Bandura’s social cognitive model, which proposes individual self-efficacy as central to the process of the adoption and maintenance of positive health behaviours. Higher self-efficacy is strongly associated with more healthful behaviours [25]. The training aims to enhance health and social care practitioners’ self-efficacy to address a range of lifestyle issues and support patients and clients to change. Empowering individuals to identify solutions and take first steps towards their goals provides mastery experiences which Bandura proposes is one mechanism for raising self-efficacy.

Healthy Conversation Skills support is delivered by trained research nurses. The trial has a team of six part-time research nurses: three who have contact with women in the intervention group and three who have contact with women in the control group. Intervention nurses receive training in Healthy Conversation Skills. These are skills that support behaviour change, thus enabling practitioners to empower women to change their own health behaviours. The training promotes the development of practitioner skills in reflecting, listening and asking ‘open discovery questions’: those that generally begin with ‘what’ or ‘how’. These questions, when used in conversation, allow a client to explore an issue, identify barriers, generate solutions and set goals. Skills in Specific, Measurable, Action-oriented, Realistic, Timed, Evaluated, Reviewed (SMARTER) goal setting are also developed during the training to enable practitioners and clients to review progress towards meeting goals at each successive meeting. As such, Healthy Conversation Skills training has been developed to incorporate the use of a range of behaviour change techniques (BCTs) [26]. These are used throughout the training sessions, and trainees are introduced to many of them explicitly in some of the training activities. The training programme is described in more detail in Table 1. Control group nurses are trained in study procedures but do not have any specific training in skills to support behaviour change and so do not use open discovery questions or support women to set goals.

**Table 1:** Healthy Conversation Skills (HCS) training description

Communication is enhanced through practitioners developing the skill of asking open-ended questions, known as Open Discovery Questions: those that generally begin with ’how’ and ’what’. Such Healthy Conversations allow a patient or client to explore an issue, identify barriers and generate solutions that can be reviewed with the practitioner. Training aims to increase self-efficacy and a sense of control of both practitioners and their patients and clients.
The five core skills are:
1. To be able to identify and create opportunities to hold Healthy Conversations
2. To use Open Discovery Questions to support someone to explore issues, barriers and priorities; problem-solve; generate solutions; and set goals for change
3. To reflect on practice and conversations
4. To spend more time listening than giving information or making suggestions
5. To support someone to make a SMARTER (Specific, Measurable, Action-oriented, Realistic, Timed, Evaluated, Reviewed) plan.
Healthy Conversation Skills training generally consists of two 3–4 hour group sessions over a week or so to allow time for practising and reflecting on skills between sessions. Training is delivered by an HCS trainer experienced in group work and behaviour change to a group of about 8 to 15 trainees. This can be followed by a period of ongoing support, which might include a phone call or face-to-face visit from one of the trainers to find out how skills are being implemented in practice. The phone call and visit allow trainees to reflect on the training, how they have implemented new skills, any barriers to their implementation and plans for continued or increased use, including embedding self and peer reflection as part of normal practice. Both follow-up activities are also opportunities to collect evaluation data to assess the effectiveness of the training in changing staff practice, using customised tools developed by the HCS team.

Once HCS nurses are trained, they are supported to embed the skills rapidly into their practice. Ongoing support is provided by the HCS trainers, who are members of the research team, during regular meetings. Every interaction between nurses and participants is recorded in a Case Report Form, and a sample of appointments for each nurse are audio-recorded and assessed in detail as part of the process evaluation. This approach will allow us to capture variations between nurses.

The objective of having a Healthy Conversation is to explore the woman’s world and support her to identify issues, solutions and goals. She is therefore supported by the trained research nurses to review these topics at each visit. Progress with any previously set goals is explored, and if women wish to change or set new/additional goals, this is entirely acceptable and is recorded by the nurses. A Case Report Form is completed for every participant by each nurse at each visit to record all goals set and the women’s experiences in working towards and achieving these goals, as well as any barriers and unsuccessful attempts to change. The women can set as many or as few goals as they wish.

During the trial, the group of women randomised to receive the behaviour change intervention will meet with a nurse trained in Healthy Conversation Skills on four occasions during pregnancy and once after birth. The control group will have the same number of contacts.

### Vitamin D supplementation

Vitamin D supplementation consists of a daily oral supplement of 1000 IU cholecalciferol. Each woman receives a single box of blister-packed capsules of vitamin D or placebo to last for the duration of her pregnancy. Each pack is individually prescribed for each participant. The trial’s pharmacist allocates a pack to that prescription, documenting both the pack number and the SPRING participant’s unique ID number. These are checked again by the research nurse, who collects them from pharmacy ready to issue to participants at the 14 week visit, and documented in the participant’s notes. The medication comes with a tear-off adhesive label which is placed in the notes as an added safeguard against error in pack allocation. A sticker is applied to the obstetric notes of women taking part in the trial in order to alert clinicians to their enrolment. An information sheet and copy of the consent form are filed in each woman’s notes. The mother (with a copy to the father) is given contact details for the study co-ordinator and research nurse and asked to inform them immediately when labour begins.

The Investigational Medicinal Product (IMP) and matched placebo are manufactured by Sharp Clinical Services (UK) Ltd, Crickhowell, Powys, Wales NP8 1DF, UK. The manufacturer has no role in the trial other than supply of the randomised IMP. As with the MAVIDOS trial, the IMP capsules contain 1000 international units (IU) of cholecalciferol (vitamin D3, supplied by Merck KGaA, Darmstadt, Germany, who also had no other role in the trial), with excipients matched to the placebo capsule [9]. Study medication (active/placebo) is supplied to the local pharmacy pre-randomised 1:1 by the manufacturer, using a computer system, and sequentially numbered for storage and dispensing. Code-break envelopes are supplied to the lead pharmacist but are not available to the investigative team. Emergency code-break access is available through the principal investigator and on-call pharmacist.

### Randomisation

Consistent with the trial’s factorial design, women are randomised to receive Healthy Conversation Skills support or to usual care and to receive a vitamin D supplement or a matched placebo. The impartial trial data manager uses block randomisation to allocate participants to receive support from a practitioner trained in Healthy Conversation Skills, or to usual care. Blocks of 4 are used, each with two 0’s and two 1’s. This ensures that for each 4 participants randomised there will be equal distribution of intervention and control participants. While is it not possible to blind staff to the allocation ﻿of Healthy Conversation Skills, the women themselves will not be told what the intervention entails and so will be blind to the intervention they are receiving. The IMP is pre-randomised by the manufacturer using a computer system. The women are blind to their vitamin D allocation, as are the research team.

### Early pregnancy

At around 14 weeks gestation women attend the research clinic at Princess Anne Hospital. The timing of this appointment can be later if they are not recruited by 14 weeks. During the appointment, an interviewer-led questionnaire is administered to gather information on parity, baseline demographic features, diet, smoking, alcohol intake, exercise, sunlight exposure, general health, medication, psychological health and wellbeing, self-efficacy and food involvement. Diet is assessed using a 20-item food frequency questionnaire (FFQ) which was developed from a larger FFQ [23]. Data from the FFQ are used to produce a standardised score (with mean 0 and standard deviation 1.0). This score has been named the ‘prudent diet score’. The 20-item FFQ gives closely comparable scores to the full FFQ and can be used in settings where administration of a longer FFQ is not feasible.

For women in the Healthy Conversation Skills intervention group, this appointment also presents an opportunity for the first Healthy Conversation with a trained research nurse. Women are asked about their lifestyle and health behaviours and what they would like to do to ensure they have healthy pregnancies and healthy babies, and to set some goals for achieving this. Details of the conversations and goals are recorded in a Case Report Form and revisited at future appointments.

Women’s height, weight, skinfold thickness (triceps, biceps, subscapular and suprailiac) and grip strength are measured. Venous blood samples are taken and stored with the aim of measuring biomarkers of quality of diet, including beta carotene and vitamin D. With participants’ consent, blood pellets are stored to allow future DNA analysis.

### 18–21 weeks gestation

Women attend at 18–21 weeks for a National Health Service (NHS) fetal anomaly scan and an additional high-resolution 3D ultrasound scan to obtain detailed measurements of their pregnancies. These scans are performed by the SPRING study sonographer. If any abnormalities are identified, women are referred to their local fetal medicine department for further management and might be excluded from this trial if the diagnosis would make it difficult for them to continue either with the IMP or the study procedures. The findings of any abnormal scans will be recorded as adverse events and thus might inform potential new hypotheses regarding the action of vitamin D in pregnancy. A pill count is performed to assess compliance with the study.

During the 18–21 weeks visit, and after the scan, the intervention research nurses hold a second Healthy Conversation with women in the Healthy Conversation Skills intervention groups to discuss their pregnancies, explore progress towards any goals they set at the 14 week visit and to set new ones if need be. Details of conversations are recorded on the Case Report Form. This forms part of the implementation component of process evaluation. Control research nurses give control group women an information sheet and confirm the timing of the 26 week phone call.

### 26 weeks gestation

At 26 weeks gestation, all women taking part in the trial receive a phone call from the research nurses. Healthy Conversation Skills intervention women are phoned by intervention nurses who hold a Healthy Conversation with them and explore progress towards their goals. Women in the normal care control group receive calls from control group nurses. The call is used to check personal details and confirm the time of their next appointment. All calls, to both intervention and control women, are audio-recorded to inform the evaluation of intervention implementation.

### 34 weeks gestation

Women attend the research clinic at 34 weeks gestation for a repeat growth and 3D ultrasound scan, questionnaire and blood tests. Those in the Healthy Conversation Skills intervention group have a Healthy Conversation with an intervention research nurse to monitor their goals and offer support. Women in the control group have contact with control group nurses and receive standard antenatal care. Questionnaires administered at this visit assess intake of foods and supplements containing vitamin D, smoking, alcohol, exercise, medications, health, well-being and self-efficacy. Venous blood samples taken at this visit are used to measure 25(OH) vitamin D, calcium, alkaline phosphatase, albumin and dietary biomarkers such as beta carotene and vitamin C. Any woman found to be hypercalcaemic (serum calcium >2.75 mmol/l) is followed up and managed appropriately. Compliance with the study medication is assessed by pill count. Mothers are given an information sheet about the neonatal DXA scan at this visit.

### Admission to hospital for labour

The study co-ordinator/research nurse is informed of any women entering labour by the NHS midwives or by the women themselves or their partners. The attending midwives collect venous umbilical cord blood (including samples for genetic analyses) and placental and umbilical cord tissue samples at delivery. The tissue samples will allow exploration of the effect of vitamin D supplementation on calcium transport across the placenta. After ensuring the infant’s hips have been assessed by a paediatrician to exclude congenital hip dislocation, a research nurse measures the baby’s length, weight, skinfolds and abdominal circumference, and arranges an appropriate time for the baby to undergo bone density assessment by DXA.

### Neonatal DXA

Infants receive DXA scans within the first 2 weeks after delivery. The reliability of DXA measurements in neonates has been demonstrated in the Southampton Women’s Survey [2]. The baby is pacified, fed, fully undressed and then swaddled in a towel before being placed on the densitometer (Hologic Discovery instrument using paediatric software (Hologic Inc., Bedford, MA, USA)). The scanner is calibrated against a spine phantom every day together with daily quality assurance and step-wedge calibration, performed as per manufacturer’s instructions (Hologic Inc., Bedford, MA, USA). If possible, the baby is scanned while still an inpatient. If this is not possible, the mother and baby return for assessment within 14 days of birth. Whole body and lumbar spine bone area, bone mineral content and bone mineral density are measured. The infant DXA assessments are associated with a low dose of radiation exposure, roughly equivalent to 2 days of background radiation in Cornwall (UK) and 7 days in other parts of the UK.

### One month after birth

Mothers and their babies are visited at home by an intervention or control nurse, depending on their allocation to Healthy Conversation Skills. Questionnaires are administered repeating the questions used at 34 weeks but also asking questions about infant feeding in order to establish whether breastfeeding has been initiated and is maintained. Mothers in the Healthy Conversation Skills intervention group also have a Healthy Conversation with a research nurse during which the mother’s goals for herself and her baby are reviewed. As before, the content of the conversation is recorded. All mothers are asked whether they consent to be contacted in the future if the study receives funding for a follow-up during childhood.

### Inclusion and exclusion criteria

#### Inclusion criteria

The inclusion criteria are as follows:

- Less than 17 weeks gestation at recruitment based on last menstrual period (LMP)
- Aged over 18 years
- Singleton pregnancy
- Aiming to give birth at local maternity (Princess Anne) hospital.

#### Exclusion criteria

The exclusion criteria are as follows:

- Known metabolic disease or chronic disease associated with bone metabolism
- Current medication likely to interfere with intrauterine growth
- Inability to give informed consent
- History of renal stores, hyperparathyroidism or hypercalciuria
- A diagnosis of cancer in the last 10 years
- Serum calcium >2.75 mmol/l.

### Timing of pregnancy

Many women do not know the exact timing of conception. Thus, gestation based on the timing of a woman’s last menstrual period (LMP) and that based on ultrasound scan often differ. Therefore, women coming for their dating scan between 8 and 12 weeks might find they are actually a few weeks earlier or later in their gestation than they thought. All assessments will be performed as closely as possible to the gestational timing outlined in the protocol. Where this is not possible, the following limits will be observed:

- Women with gestations between 8 and 17 weeks can be included in the trial
- Dating scans take place between 8 and 12 weeks
- Nuchal translucency scans cannot be performed before 11 weeks, and so women with pregnancies of earlier gestation might be asked to return at 11 or 12 weeks
- Early pregnancy visits will be performed as closely as possible to that time but will be acceptable until the anomaly scan at 18–21 weeks
- The anomaly scan is performed between 18 and 21 weeks
- The 34 week scan will be performed between 33 and 36 weeks but, if delayed, can be performed up to delivery

### Sample size and power calculation

Our power calculation uses an alpha of 0.025 taking into account the two primary outcomes of maternal vitamin D and maternal diet quality. With 150 participants in each arm of the trial (equivalent to 300 participants per arm in each comparison between HCS and routine care and between vitamin D and placebo) there is 80 % power at an alpha of 0.025 to detect a 0.25 standard deviation difference in both diet quality score and vitamin D (approximately 3 nmol/l 25(OH)) between groups. Allowing for a 20 % drop-out rate, we aim to recruit 188 women to each arm of the four arms of the trial in order to achieve 150 women in each arm. Pilot data suggest that around 40 % of women who are approached will agree to participate in the study, and so initial invitations will be issued to 1250 women. Experience in the MAVIDOS trial indicates that women will be recruited at a rate of 5–8 per week, suggesting that recruitment will take up to 3 years.

### Statistical analyses

We will perform a complete case analysis for each outcome using *t* tests to compare the two primary outcomes between the relevant intervention arms. Multiple linear regression will be used to adjust for any differences identified at baseline. Similar analyses will be conducted for the secondary outcomes. The neonatal bone and body composition analyses will include stratification by season of birth in light of recent findings from the MAVIDOS trial [10]. We will also conduct exploratory analyses of the interaction between HCS and vitamin D on both primary outcomes. For binary outcomes, chi-squared tests or Fisher’s exact test for rare outcomes will be used. To adjust for imbalances at baseline, logistic regression will be used, or for rare outcomes, binary regression or Poisson regression with robust variance.

### Process evaluation

In accordance with Medical Research Council (MRC) guidance, our process evaluation will assess implementation, mechanisms of action and context [27]. It runs throughout the trial. We have involved stakeholders, including maternity and public health practitioners and members of a lay advisory panel, in the development of a logic model which describes the way in which the intervention processes will bring about change in the outcomes. Implementation is being assessed through observation and recording of staff interactions with participants and includes an assessment of Healthy Conversation Skills research nurses’ competence in the skills they have been taught and their confidence in the use of these skills. Recruitment and retention of participants will be monitored throughout the trial. Mechanisms of action will be assessed through interviews with women to find out their experiences of taking part in the trial and, in particular, their experiences of contact with nurses trained in Healthy Conversation Skills. We will also examine intermediate outcomes, as we hypothesise that change in diet will be facilitated by improvements in women’s self-efficacy. In assessing context, we will aim to identify any factor that might act as a barrier or facilitator to intervention implementation or effects and will carry out detailed mapping of local services and national policy that might influence the delivery and efficacy of the intervention.

### Ethical considerations

Information sheets are given to participants outlining the procedures that they will undergo, and plain English is used to facilitate participants’ understanding of the study. Participants also have opportunities, at each follow-up appointment, to discuss any concerns with study staff. The infant DXA assessments are associated with a low dose of radiation exposure, roughly equivalent to 2 days of background radiation in Cornwall (UK) and 7 days in other parts of the UK including Southampton. The dose of vitamin D supplementation has been chosen to bring women just into the normal range (>50 nmol/l circulating 25-hydroxy vitamin D), to avoid elevating to supranormal levels. This approach is consistent with that taken in the MAVIDOS trial of vitamin D supplementation during pregnancy [10]. Previous observational studies in Southampton have suggested low risk of adverse effects from vitamin D supplementation: data from the Princess Anne Cohort study showed a small excess of atopic asthma in children born to mothers with the highest levels of vitamin D in pregnancy [28]. However, numbers of children affected were lower than expected from general population prevalence, and the confidence intervals around the association were wide. No associations between atopy in infancy and high levels of maternal vitamin D have been shown in the Southampton Women’s Survey, and other studies have suggested neutral or negative associations [29–31].

Blood and tissue samples will be processed for storage on the day of collection and will be stored in freezers in the MRC Lifecourse Epidemiology Unit, Southampton General Hospital. They will be stored until all analyses are completed. Professor Cyrus Cooper, Director of the Unit and chief investigator for the SPRING trial, will have custodial responsibility, and the samples will be stored in compliance with Human Tissue Authority (HTA) regulations.

There is a potential risk of distress with any interview. Staff will be trained in interviewing techniques and will terminate an interview in the rare event that questions cause participants distress. If a participant should become distressed, she will be reminded of the helpline number and will be referred to support services, a list of which will be available in the research clinic.

### Reporting of adverse events

#### Anticipated adverse events

Some pregnancy-associated complications are expected to arise spontaneously during the study and are not associated with vitamin D supplementation. These include cholestasis of pregnancy, pregnancy-induced hypertension, gestational diabetes, pre-eclampsia, eclampsia, premature labour or delivery, instrumental delivery, Caesarean section, miscarriage or stillbirth.

#### Adverse drug reactions and adverse drug events

Any adverse event which might be linked in any way to vitamin D supplementation is immediately reported to the sponsor. A detailed written report on the event is produced. The principal investigator decides whether to expedite reports of adverse events felt to be unrelated to the IMP. A record of all serious adverse events is kept in the trial site file.

The sponsor keeps detailed records of all adverse events reported to them by the principal investigator. These records may then be sent to the licensing authority if required.

#### Suspected unexpected serious adverse reactions

The sponsor ensures that all relevant information about suspected unexpected serious adverse reactions which are fatal or life-threatening that occur during the course of the trial are reported as soon as possible to the Medicines and Healthcare products Regulatory Agency (MHRA) and the relevant ethics and data monitoring committees. This is done no later than 7 days after the sponsor was first made aware of the reaction. Any additional relevant information is sent to the MHRA within 8 days of the initial report.

Suspected unexpected serious adverse reactions that are not fatal or life-threatening are reported to the MHRA and the relevant ethics/data monitoring committee no later than 15 days after the sponsor becomes aware of them.

### Data collection and management

To guarantee the validity and integrity of trial design and conduct, two committees are being established. The trial steering committee is overseeing trial design and implementation. An independent data monitoring committee is overseeing the data collection process.

### Regulatory aspects

The study has received approval from the MHRA, Southampton and South West Hampshire Research Ethics Committee, University Hospital Southampton R and D (sponsor) and the UHS Data Protection Office. The IMP and placebos are manufactured in accordance with Good Manufacturing Practice (GMP) regulations. The study is conducted in compliance with the Research Governance Framework for Health and Social Care, the Medicine for Human Use (Clinical Trials) Regulation 2004 and with Good Clinical Practice (GCP). Indemnity has been provided through the University Hospital Southampton NHS Foundation Trust (sponsor) and the University of Southampton.

## Discussion

Intervening to improve the nutritional status of pregnant women will optimise the growth and development of their babies, which will in turn reduce their risk of cardiovascular disease and obesity when they reach adulthood [1]. The combination of nutrient supplementation and behavioural intervention being tested in SPRING represents a multi-component approach that has the potential to address vitamin deficiencies and to improve general quality of diet. Research suggests that both are important in improving outcomes for women and their babies. The factorial design of this trial has the advantage of enabling each intervention to be tested separately as well as allowing exploration of the synergistic effect of both interventions on women’s diets and vitamin D levels.

### Trial status

The SPRING trial is ongoing.
